# Clinical Performance Evaluation of Demographic-Based Liver Volumetry

**DOI:** 10.3390/life16091463

**Published:** 2026-09-01

**Authors:** Yasaman Anbari, Benjamin P. Lopez, Armeen Mahvash, Ali Yousefi, Srinivas C. Kappadath

**Affiliations:** 1 Department of Imaging Physics, The University of Texas MD Anderson Cancer Center, 1155 Pressler St. Unit 1352, Houston, TX 77030, USA; yanbari@mdanderson.org (Y.A.); bplopez@mdanderson.org (B.P.L.); 2 Department of Biomedical Engineering, The University of Houston, Houston, TX 77204, USA; aliyousefi@central.uh.edu; 3 Department of Interventional Radiology, The University of Texas MD Anderson Cancer Center, Houston, TX 77030, USA; armeen.mahvash@mdanderson.org

**Keywords:** liver volumetry, resection, postoperative hepatic insufficiency, future liver remnant, calibration

## Abstract

Patient demographic-based standard liver volume (SLV) models are widely used to estimate patient-specific hepatic metabolic demand and support future liver remnant assessment after hepatectomy. These formulae rely on anthropometric variables such as weight, height, and body surface area; however, their accuracy for individual patients remains uncertain. In an exploratory single-center study, we evaluated ten published SLV formulae and one in-house multivariable regression model against CT-derived liver volumes as the anatomic reference standard in 100 patients with normal livers, using Bland–Altman analysis. Mean percentage bias ranged from −20% to +11%, with only 4–32% of cases achieving prediction errors within ±5%, and the 95% limits of agreement were wide for every model, spanning from ±25% to ±36% around the mean bias. Because percentage bias is expressed relative to the measured volume, the width of the limits of agreement was almost entirely determined by calibration (r = 0.99 with signed mean bias). Once this scaling was removed, the ten published formulae were indistinguishable, with calibration-adjusted limits of agreement of ±31.3% to ±32.7% (mean ±32.0%, SD 0.40). Rescaling each formula by a single multiplicative constant (0.924 to 1.282) reduced mean bias to between +1.2% and +3.6% and converged the limits of agreement to ±32.4% to ±33.1%, raising the proportion of patients estimated within ±10% from 13% to 54% for the most poorly calibrated formula; refitting both slope and intercept gave no further improvement. The in-house model, using five predictors fitted within this cohort, reached ±29.4% and was the only specification below this floor. In this exploratory single-center cohort, prediction error was wide across the observed range and showed no dependence on predicted liver volume. The limitations for SLV appear to lie in the demographic predictors rather than in the reference measurements. Therefore, future work should focus on predictors that capture information beyond body size, such as measures of body composition or liver function.

## 1. Introduction

Liver volumetry plays a pivotal role in hepatology and surgical planning, offering quantitative insights that directly influence clinical decision-making. Major hepatic resection remains the cornerstone of curative therapy for primary and metastatic liver malignancies. Patient-specific healthy liver volume estimates are used by clinicians to assess the organ’s capacity to withstand surgical interventions, such as hepatic resections and transplantations. Standard liver volume (SLV) serves as a foundational parameter in hepatic resections and transplantation, providing a reference value to reflect the hepatic metabolic demands for a specific patient [1]. The concept of “Standard Liver Volume” was first introduced by Kawasaki et al. in 1992, within the context of living donor liver transplantation, where they observed that transplanted grafts gradually converged to the recipient’s SLV calculated based on body surface area (BSA), regardless of whether the initial graft was smaller or larger than the recipient [2]. Over the next decade, several predictive formulae were developed to estimate SLV more precisely. In 1995, Urata et al. established the first quantitative formula for SLV based on BSA, derived from CT-measured liver volumes in a Japanese population [3]. In 1999, Heinemann et al. [4] created the first SLV formula tailored to the Caucasian population using autopsy data. Later, in 2002, Vauthey et al. introduced another BSA-based SLV formula that has since been widely adopted in Western clinical practice [5,6]. Together, these developments have positioned SLV as a foundational metric in transplant planning and postoperative assessment.

In living donor liver transplantation, SLV is crucial for determining graft adequacy and ensuring donor safety, often through calculations such as the graft-to-recipient weight ratio or the future liver remnant (FLR). Postoperative outcomes are critically dependent on the functional capacity of the FLR because insufficient FLR is strongly associated with postoperative liver failure, a leading cause of morbidity and mortality after hepatectomy [7]. To mitigate this risk, preoperative assessment of the total liver volume (TLV) and calculation of the FLR/TLV ratio have become standard practice. Current guidelines recommend minimum FLR/TLV thresholds of 20–25% for patients with normal livers, ≥30% in the setting of steatosis or chemotherapy-associated liver injury, and ≥40% for cirrhotic livers [8]. Accurate estimation of SLV is essential for assessing surgical candidacy and minimizing the risk of postoperative liver failure. The use of SLV rather than relying solely on the patient’s actual CT-measured liver volume is important because the measured liver volume may not always represent the patient’s ideal functional liver requirement. In patients with liver tumors, cirrhosis, steatosis, hypertrophy, atrophy, or prior treatment-related changes, the actual CT-derived liver volume can be altered by disease burden or compensatory remodeling. SLV provides a standardized estimate of the expected or required healthy liver volume based on patient-specific demographic characteristics, allowing clinicians to compare the observed liver volume or future liver remnant against the individualized referenced volume.

Despite the availability of numerous SLV models (Table 1), several critical gaps remain in our current understanding of SLV prediction models, including the accuracy of the SLV models and the behavior of the errors across the full spectrum of liver sizes. The aforementioned studies relied on statistical metrics, such as the coefficient of determination (R^2^), to report on the adequacy of the SLV models, but failed to report on their accuracy. Furthermore, clinical relevance was seldom validated, leading to potential misapplication in real-world scenarios. Underestimation of the SLV may lead to insufficient residual liver mass after surgery, significantly increasing the risk of post-hepatectomy liver failure, a life-threatening complication. In contrast, overestimation of SLV may prompt conservative decisions, such as deferring potentially curative resection/surgery or rejecting suitable donor candidates. While both miscalculations are problematic, the underestimation of SLV poses a greater clinical risk due to its direct association with operative failure and postoperative morbidity. Another key limitation was that most prior publications did not clearly define the demographic range (e.g., weight, BSA, age) in which the formulae are applicable, thereby increasing ambiguity in their applicability to site-specific patient populations. Lastly, a widespread issue is the lack of availability of independent liver volumetry datasets for model validation.

These limitations highlight the need for a more robust, clinically oriented, and externally validated approach to liver volume prediction methods that are transparent in scope, report on model accuracy, accurate in diverse populations, and aligned with surgical decision-making thresholds. The objectives of this work were to (1) independently characterize the performance of published SLV models in terms of model accuracy and precision, (2) develop and characterize an in-house multivariable regression model based on demographic data to predict liver volume, and (3) evaluate that model in a held-out test cohort of patients from an earlier time period in 2015 who were not used in its training.

## 2. Material and Methods

### 2.1. Patient Data Selection

The patient cohort consisted of stage 2 lung cancer patients who underwent whole-body PET/CT or abdominal CT imaging between 2015 and 2016 at our institution, with the complete liver imaged within the scan field of view (FOV). All scans were obtained before any oncological treatment and were performed for non-hepatobiliary indications. Inclusion criteria additionally required the absence of focal hepatic lesions, no history of malignancy other than the index lung cancer, no evidence of advanced fibrosis or cirrhosis, no history of hepatic surgery, and no signs of cholestasis.

We used CT images of normal liver from 135 patients (221 screened) scanned at our institution to develop and characterize an in-house multivariable regression model. Out of 135 patients, 100 liver scans (57 females and 43 males) were used as the training cohort to develop the model, while 35 liver scans (21 females and 14 males) were used as the test cohort to validate and characterize model performance. For all patients, sex, age, height, and weight at the time of CT were recorded. All included patients were of white ancestry. Patients of other self-reported race or ethnicity were excluded because liver volume at a given body size has been reported to differ between ethnic groups, and a homogeneous cohort avoids confounding of the volume–anthropometry relationship by ethnicity.

The study was conducted in accordance with the Declaration of Helsinki and was approved by the University of Texas MD Anderson Institutional Review Board. Written informed consent for use of normal liver images was waived (DR09-0025; approved October 2024) because the analysis was retrospective, used only imaging and anthropometric data acquired for routine clinical care, and involved no additional intervention or contact with patients; all data were de-identified before analysis.

#### 2.1.1. CT Liver Volumes

CT images were obtained as part of either whole-body PET/CT or diagnostic CT examinations. Images were acquired during free breathing with a slice thickness of 2 or 3.75 mm. Because the CT component of PET/CT was acquired during free breathing, respiratory motion may have caused minor blurring of the hepatic margins. This effect was expected to influence measurement precision but not produce systematic measurement bias. The segmented liver volume included the major intrahepatic vessels.

Liver contours for patients were automatically generated using a commercially available AI-based segmentation platform (automated), Contour ProtégéAI+, MIM software version 7.3.7 (MIM Software Inc., Cleveland, OH, USA). Volumes were confirmed by a board-certified radiologist to make sure that the generated contours contained the whole liver. The segmented liver volumes obtained from CT imaging were used as the reference for evaluating SLV models throughout this study. The use of CT volumetry as a reference standard is supported by prior validation studies comparing CT-based virtual liver volumes with intraoperative measurements of resected liver specimens, graft volumes, or graft weights, which have demonstrated strong correlations [9]. Therefore, CT-derived volumetry provides a clinically relevant patient-specific anatomic reference for liver volume assessment.

#### 2.1.2. SLV Models

A summary of the 10 previously published SLV models and their parameters evaluated in this work is provided in Table 1.

**Table 1:** Summary of demographic-based SLV prediction models evaluated in this study showing the model input parameters and the corresponding regression equation used to estimate liver volume (in mL). (^a^ Sex is coded as 1 for male and 0 for female; ^b^ Dubois and Dubois formula [10]; ^c^ Mosteller formula [11]) * Age = 1: For patients < 40 years old Age = 2: For patients 40–60 years old Age = 3: For patients > 60 years old.

Model Name	Model Parameters	SLV (mL) Equation
Chan [12]	Weight (kg), Sex	SLV = (12.3 × weight) + (51 × sex) + 218
Fu-Gui [13]	Weight (kg)	SLV = (11.508 × weight) + 334.024
Hashimoto [14]	BSA (m^2^) ^b^	SLV = (961.3 × BSA) − 404.8
Heinemann [4]	BSA (m^2^) ^b^	SLV = (1072.8 × BSA) − 345.7
Lin [15]	Weight (kg), Height (cm)	SLV = (12 × weight) + (13 × height) − 1530
Poovathumkadavil [16]	Weight (kg)	SLV = (12.26 × weight) + 555.65
Vauthey-BSA [5]	BSA (m^2^) ^c^	SLV = (1267.28 × BSA) − 794.41
Vauthey-weight [5]	Weight (kg)	SLV = (18.51 × weight) + 191.80
Yu [17]	Weight (kg), Height (cm)	SLV = 21.585 × weight^0.732^ × height^0.225^
Yuan [18]	BSA (m^2^) ^b^, Age *	SLV = (949.7 × BSA) − (48.3 × age) − 247.4 *
Multivariable Regression model (this work)	Weight (kg), Height (cm), Sex ^a^, Age (years), (Weight-77) × Sex ^a^	$\begin{array}{c} SLV=197.39+8.68\times weight+6.10\times Height-\\ 6.14\times Age+50.96\times sex+11.30\times (weight-77)\times sex \end{array}$

### 2.2. In-House Demographic-Based Liver Volumetry Model

#### 2.2.1. Predictor Coding

Sex was coded 1 for male and 0 for female. Weight, height, and age were entered into all models as continuous variables. S Age was also entered as a tertile indicator (cut points 66 and 73 years) in one candidate specification; these indicators do not appear in the final model. Body surface area was calculated with the Du Bois formula for all in-house models. All specifications were fitted to the same 100 complete cases, so that F-tests and cross-validated errors are directly comparable.

#### 2.2.2. Candidate Model Structure

Candidate models were specified in advance in four tiers, each addressing a distinct question about model structure rather than forming an unordered set of alternatives. The tiers are ordered, but not every comparison between them is nested, and the method of comparison therefore differs by step.

Tier I—body size alone. Liver volume was modeled on a single size descriptor, either weight or body surface area, corresponding to the form used by most published SLV formulae. These two models are not nested within one another and were compared by cross-validated prediction error only. Because BSA is itself a function of weight and height, the BSA model already carries height information that the weight model does not; the two Tier I branches are consequently not equivalent in information content, and the incremental contribution of height in Tier II is correspondingly smaller for the BSA branch.

Tier II—additive anthropometric terms. Height, sex, and age were added to the Tier I weight model in that fixed, prespecified order, to establish whether each contributed information beyond body size alone. Each step is nested within the one preceding it and was tested by a nested F-test. Because sequential tests depend on the order of entry, the order was fixed in advance.

Tier III—functional form of the size term. Retaining the Tier II covariates, the linear weight term was replaced in turn by a quadratic, a square-root, and an allometric (log–log) specification, to test whether the volume–size relationship departs from linearity. Only the quadratic specification is nested within the linear model—the linear model being the restriction that the squared-weight coefficient equals zero—and only that comparison was tested by an F-test. The square-root and allometric specifications are not nested within the linear model and were compared on cross-validated prediction error alone. In the allometric specification, liver volume and weight were both log-transformed, and height, sex, and age were retained on their original scales.

Tier IV—interaction. A weight × sex interaction was added to the Tier II model to test whether the slope relating weight to liver volume differs between the sexes. This term was prespecified on physiological grounds: at equal body weight, women carry a higher proportion of adipose tissue than men, so a given increment in weight corresponds to a smaller increment in lean tissue. A weight × height interaction was also fitted, but as an exploratory comparison rather than a prespecified hypothesis. The interaction was formed from mean-centered weight, so that the lower-order coefficients describe effects at the sample mean weight of 77.0 kg rather than at a weight of zero, and to improve the numerical conditioning of the design matrix, centering did not alter the interaction coefficient, its standard error or its *p*-value. Because tests of interaction are less powerful than tests of main effects, the interaction is reported as an estimate with a 95% confidence interval alongside the sex-specific weight slopes rather than as a *p*-value alone.

#### 2.2.3. Model Comparison

Nested comparisons—Tier I to Tier II, linear to quadratic within Tier III, and Tier II to Tier IV—were tested by F-test, and are reported as descriptive rather than confirmatory, since twelve specifications were examined and no adjustment for multiplicity was applied. Non-nested comparisons—weight versus BSA in Tier I, and the square-root and allometric specifications in Tier III—cannot be assessed by F-test and were compared on cross-validated prediction error only.

All specifications were ranked by prediction error from 200 repetitions of five-fold cross-validation, using identical resampling splits for every model so that values are directly comparable. Predictions from models fitted on the logarithmic scale were back-transformed and corrected with Duan’s smearing factor, estimated separately within each training fold, so that all models were compared on the milliliter scale.

### 2.3. Data Analysis

All statistical analyses were performed in Python version 3.12.11 (64-bit) under Windows 11, using NumPy 2.4.3, pandas 3.0.2, SciPy 1.17.1, scikit-learn 1.8.0, and Matplotlib 3.10.8.

The performance characterization of SLV models, including 10 published and 1 in-house, was assessed against CT-derived liver volumes. For each SLV model, Bland–Altman analysis was conducted to assess the agreement between predicted and actual liver volumes via calculation of the mean percentage bias (Equation (1)), mean absolute percentage bias (MAPB), and 95%-LOA (Equation (2)) as shown below:

(1) $$b_{i}=\frac{{SLV}_{i}-V_{i}}{V_{i}}\times 100$$

where ${SLV}_{i}$ is the predicted and $V_{i}$ the CT-measured liver volume, and,

(2) $$LOA=b\bar{b}\pm Z_{\alpha /2}S_{b}$$

where Z_a/2_ = 1.96 and S_b_ is the standard deviation of the percentage bias [19]. Limits of agreement are reported throughout as the half-width, 1.96·$S_{b}$.

Calibration was separated from precision. Because $b_{i}$ is expressed relative to Vi, the spread of the percentage differences is proportional to the calibration of the formula:

(3) $$1.96\cdot S_{b}=\left(1+\bar{b}/100\right)\times LOA\_adj$$

so a formula that underestimates liver volume will show narrower limits of agreement purely as a consequence of scale, and limits of agreement cannot be compared across formulae that differ in mean bias. Rearranging Equation (3) gives a measure of precision that is independent of calibration:

(4) $$LOA\_adj=1.96\cdot S_{b}/\left(1+\bar{b}/100\right)$$

Second, each formula was recalibrated by a single multiplicative constant, defined as the median ratio of measured to predicted volume across the cohort:

(5) $$c=m{edian}_{i}\left(V_{i}/{SLV}_{i}\right)$$

The same constant was applied to every patient; the median was used rather than the mean to limit the influence of the two largest livers. Each prediction was then rescaled as

(6) $${SLV}_{i*}=c\times {SLV}_{i}\;$$

Mean bias, limits of agreement, and the proportions of estimates within ±5%, ±10%, and ±20% were recalculated by substituting SLVi_*_ for *SLVi* in Equation (1). Because Equation (6) multiplies every coefficient of a formula by the same factor, it changes the calibration of the predictions while leaving the functional form, the predictors, and the rank ordering of predictions unchanged.

As a sensitivity analysis, both parameters of each formula were refitted to the cohort by ordinary least squares,

(7) $$\hat{V}i=\alpha +\beta \;{SLV}_{i\;}$$

to establish whether refitting both the slope and the intercept reduced the residual scatter beyond what Equation (6) achieved.

The in-house model was evaluated in a test cohort of 35 patients distinct from the training cohort. No patient contributed to both samples and no parameter of the model was re-estimated in this cohort. The coefficients derived from the training cohort were applied unchanged, so that every prediction is genuinely out-of-sample.

To further investigate differences between SLV models, a recalibration (bias-correction) of the published SLV models was performed. The purpose of recalibration was to separate the two components of disagreement: the systematic bias offset, which a model-specific scaling factor can remove, and the residual scatter or variance, which cannot be corrected. Rescaling by a single constant removes the systematic offset in these data; the scatter that remains is therefore the component of disagreement not attributable to calibration. Refitting of both slope and intercept (Equation (7)) was performed to establish whether a second free parameter reduced that residual scatter further. Recalibration constants were estimated within the present cohort and are reported to isolate calibration from precision. Recalibration was performed for investigation of performance rather than as a proposed correction.

Agreement metrics for the in-house model were calculated from out-of-fold predictions obtained by 200 repetitions of five-fold cross-validation, so that no patient contributed to the model used to predict them; the ten published formulae were applied directly.

To further compare model performance, we calculated the proportions of predictions falling within prespecified absolute percentage-error bands of ±5%, ±10%, and ±20% relative to the CT-measured liver volume. The ±5% threshold was selected based on consultation with an experienced interventional radiologist (AM), who considered deviations of this magnitude unlikely to affect clinical interpretation. The ±10% threshold was included as a more stringent benchmark of prediction accuracy. The broader ±20% threshold was selected based on previous liver-volumetry literature. Salvalaggio et al. reported discrepancies of approximately 20% between preoperatively estimated graft volume and intraoperative measurements of the extracted liver, demonstrating the magnitude of error that may occur in clinical volumetry [20]. In addition, Yoshizumi et al. classified estimated liver weights that differed by 15% from the actual liver weight as acceptable [21]. We recognize that the ±5%, ±10%, and ±20% tolerance bands are not universally accepted tolerance levels. They are better interpreted as descriptive bands for error distribution rather than clinically validated decision thresholds.

The performance characterizations of SLV models are reported in terms of their overall model accuracy and precision using Bland–Altman analysis using CT-based liver volumes as the ground truth.

## 3. Results

### 3.1. Patient Cohort Characteristics

The baseline characteristics of the 135 patients included in this study are shown in Table 2, where Q1–Q3 refers to the interquartile range.

### 3.2. Bland–Altman Analysis of the Normal Liver

**Figure 1:**
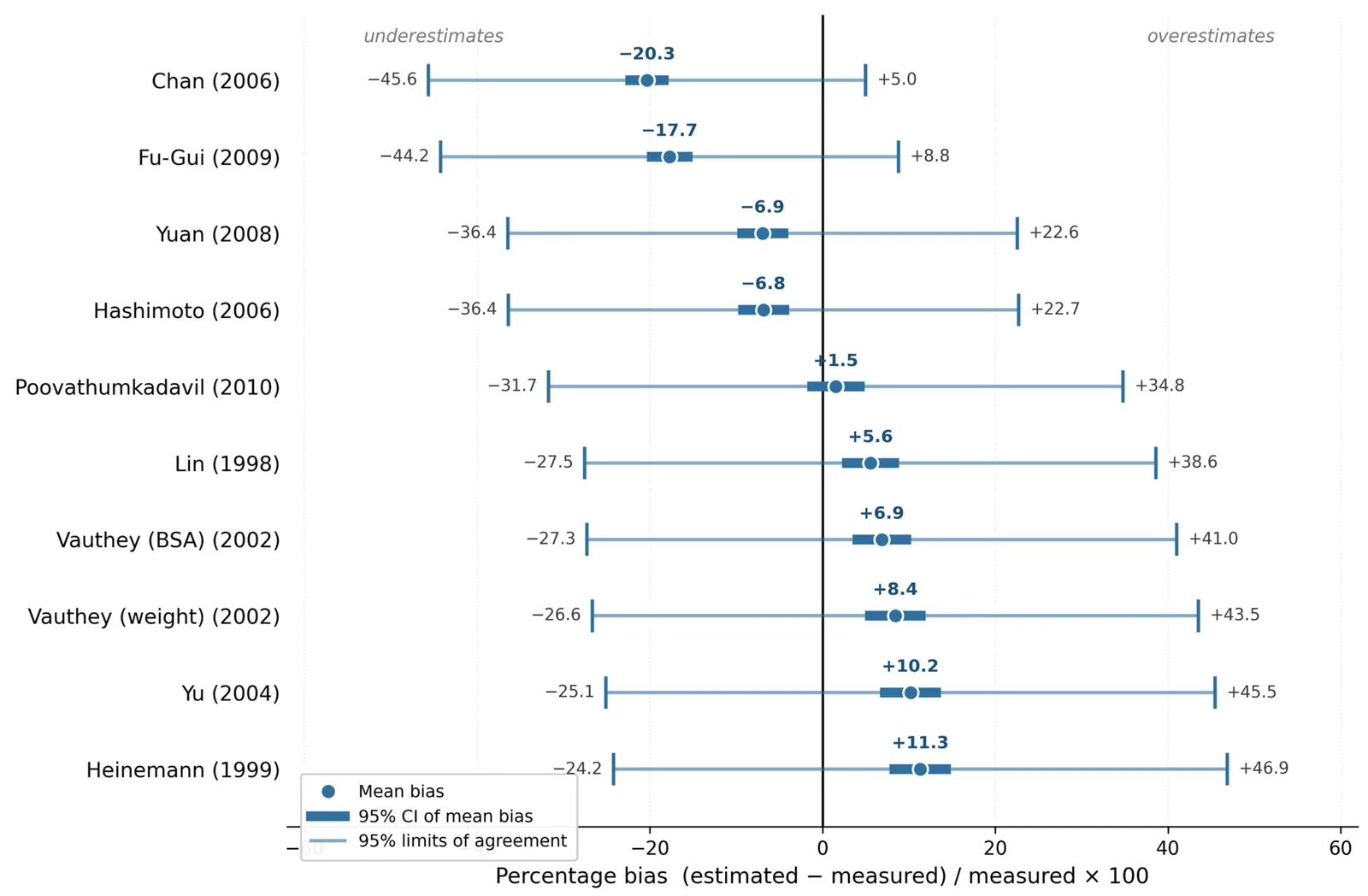
Comparison of mean percentage bias and 95%-LOA across 10 SLV models in 100 patients with normal liver, demonstrating different levels of accuracy and performance across the SLV models [4,5,12,13,14,15,16,17,18].

Bland–Altman analysis was performed for all ten published SLV models; mean percentage biases and 95% limits of agreement are summarized in Figure 1. Mean percentage bias ranged from −20.3% (Chan) to +11.3% (Heinemann). Four models systematically underestimated liver volume (Chan −20.3%, Fu-Gui −17.7%, Yuan −6.9%, Hashimoto −6.8%) and six overestimated it (Poovathumkadavil +1.5%, Lin +5.6%, Vauthey-BSA +6.9%, Vauthey-weight +8.4%, Yu +10.2%, Heinemann +11.3%). Only the Poovathumkadavil model achieved a mean bias within ±5%, while six models fell within ±10%.

Limits of agreement were wide for every model, spanning ±25.3% to ±35.5% about the mean bias, corresponding to total widths of 50.6 to 71.1 percentage points. Consequently, no model classified more than 56% of patients within ±10% of the measured volume (Lin, 56%; Vauthey-BSA, 51%; Poovathumkadavil and Vauthey-weight, 50%), and the two most biased models classified fewer than one in six (Chan, 13%; Fu-Gui, 15%). Small mean bias and narrow limits of agreement were not achieved together by any model. The narrowest limits of agreement were those of Chan (±25.3%), the model with the largest systematic bias, whereas the model centered closest to zero had relatively wide limits (Poovathumkadavil ±33.3%). Judged by the proportion of patients estimated within ±10%, the Lin model performed best overall, followed by Vauthey-BSA.

### 3.3. In-House Demographic-Based Liver Volumetry Prediction Model

#### Univariable Associations

Liver volume correlated most strongly with weight and body surface area, followed by height and sex. Age was inversely associated with liver volume (Table 3).

### 3.4. Model Selection

The model with the lowest mean cross-validated RMSE included weight, height, sex, age, and a weight × sex interaction (CV RMSE 207.9 mL; in-sample R^2^ 0.744). Adding a quadratic weight term to this model gave 209.9 mL; the same model without age gave 214.1 mL, weight alone gave 231.4 mL and BSA alone gave 230.5 mL (Table 4).

Cross-validated prediction error varied little across the twelve specifications, ranging from 207.9 mL for the selected model to 234.6 mL for the least accurate, a total spread of approximately 13%. The selected model gave the lowest error, followed closely by the two quadratic specifications (209.9 and 211.5 mL) and by the same model without age (214.1 mL); the additive models without any interaction ranged from 225.3 to 227.8 mL, and the single-predictor models from 230.5 to 231.4 mL. The margin separating the leading specifications was therefore narrow, and the final model was retained on grounds of parsimony and interpretability rather than on a demonstrated advantage in prediction.

Nested F-tests supported the addition of height to the weight-only model (F_1,97_ = 6.97, *p* = 0.010) and of the weight × sex interaction to the Tier II additive model (F_1,94_ = 18.17, *p* < 0.001). Adding sex as a main effect gave no improvement (F_1,96_ = 0.05, *p* = 0.824), and age was of borderline significance (F_1,95_ = 3.90, *p* = 0.051). Within Tier III, replacing the linear weight term with a quadratic improved fit (F_1,94_ = 16.07, *p* < 0.001), although this specification did not achieve lower cross-validated error than the selected model (Table 4). Because twelve specifications were examined, these tests are reported as descriptive rather than confirmatory.

### 3.5. Final Model

All variance inflation factors were below Section 3.3, indicating no problematic collinearity (Table 5). Because the interaction was constructed from mean-centered weight, the sex main effect represents the male–female difference at the mean weight of 77.0 kg and should not be interpreted as an overall sex effect.

The complete fitted model, showing the centering of weight within the interaction term, is:

Expanding this gives two sex-specific equations:

**Women:**

$$Liver\;Volume\;\left(mL\right)=197.4+8.68\times weight\;(kg)+6.10\times Height\;(cm)-6.14\times Age$$

**Men:**

$$Liver\;Volume\;\left(mL\right)=-621.5+19.97\times weight\;(kg)+6.10\times Height\;(cm)-6.14\times Age$$

### 3.6. Clinical Performance Evaluation of the SLV Models

We compared the performance of all eleven SLV models evaluated, including the ten previously published and one multivariable regression SLV model. The key performance metrics evaluated are summarized in Table 6. Among the demographic-based SLV models, only the Poovathumkadavil model achieved a mean percentage bias within ±5% (+1.5%), though its limits of agreement (±33.3%) were no narrower than those of the other formulae once calibration was accounted for. In contrast, the SLV models proposed by Chan, Fu-Gui, Hashimoto, and Yuan exhibited larger systematic biases (−7% to −20%), indicating reduced predictive accuracy.

The in-house multivariable regression model achieved a mean percentage bias of 1.8%, a mean absolute percentage bias of 11%, and limits of agreement (±29.9%). In terms of clinical accuracy, 58% of multivariable regression model estimates fell within ±10% of the true liver volume and 88% within ±20%.

Across the ten published formulae, the half-width of the limits of agreement was almost perfectly correlated with signed mean bias (r = 0.99). Because percentage bias is expressed relative to the measured volume, its spread scales with the calibration of the formula, so this relationship follows from the definition of the metric rather than from any property of the models. Once the scaling was removed, the ten formulae were indistinguishable, with calibration-adjusted limits of agreement of ±31.3% to ±32.7% (mean ±32.0%, SD 0.40) (Table 6).

Rescaling each formula by a single multiplicative constant (0.924 to 1.282) confirmed this directly: mean bias fell to +1.2% to +3.6% and the limits of agreement converged to ±32.4% to ±33.1%. The proportion of patients estimated within ±10% rose from 13% to 54% for Chan and from 15% to 52% for Fu-Gui, while the best-calibrated formulae were unchanged (Lin 56% to 55%). In this cohort, the differences between the published formulae are therefore attributable to a single multiplicative calibration factor, and no reparameterization reduced the residual scatter below approximately ±32%. Whether this represents a general property of demographic SLV models or a feature of this particular population cannot be determined from a single-center sample of 100 patients.

The in-house model was the only specification below this floor, with calibration-adjusted limits of agreement of ±29.4%.

### 3.7. Evaluation of the In-House Model

In the test cohort of 35 patients, the in-house model showed a mean percentage bias of +5.4% and a mean absolute percentage bias of 10.9%, with a median absolute percentage bias of 10.4%. The 95% limits of agreement were −19.0% to +29.8%. On the milliliter scale, root mean squared error was 183.9 mL and mean absolute error 150.3 mL. Predictions correlated with measured volumes at r = 0.898, and 34%, 49%, and 86% of estimates fell within ±5%, ±10%, and ±20% of the measured volume, respectively. The mean percentage bias in the test cohort was +5.4% (95% CI +1.1 to +9.7), higher than the +1.8% observed in the training cohort, though the difference between cohorts was not significant (*p* = 0.096), and the 95% confidence interval for the test limits of agreement (±18.6% to ±30.2%) included the training value; with 35 patients, performance is best described as consistent rather than improved.

## 4. Discussion

This study aimed to systematically assess the accuracy and critically evaluate the clinical reliability of ten previously published SLV models and one newly developed model based on patient-specific demographic data, using CT-derived liver volumes as the reference standard. Earlier validation studies have relied largely on correlation analysis, such as the coefficient of determination (R^2^), without reporting measures of accuracy. Because correlation is a group-level property, a high R^2^ does not imply accurate prediction for an individual patient. In one patient in our cohort, a 117 kg man of 178 cm with a CT-measured liver volume of 2317 mL, estimates from the ten formulae ranged from 1679 to 2356 mL, corresponding to errors of −28% to +2%. Patient-specific liver volume prediction is particularly critical in liver transplantation and resection, where accurate estimation is essential to prevent complications and to safeguard both donor and recipient outcomes. To address this gap, we applied Bland–Altman analysis and additional accuracy metrics: mean percentage bias, mean absolute percentage bias, 95% limits of agreement, and the proportion of cases within error ranges of ±5%, ±10% and ±20%.

The ten formulae are clinically interchangeable because, once the artifactual relationship between bias and limits of agreement is removed, their precision is indistinguishable. Because percentage bias is expressed relative to the measured volume, the standard deviation of the percentage differences scales with one plus the mean fractional bias, so a formula that underestimates by 20% will show limits of agreement approximately 20% narrower for that reason alone. This accounts for the near-perfect correlation between the width of the limits of agreement and the signed mean bias across the ten formulae (r = 0.99), of which the Chan model is the clearest example: the narrowest limits (±25.3%) combined with the largest bias (−20.3%) and only 13% of patients estimated within ±10%. Once the scaling is removed, the ten formulae are indistinguishable, with calibration-adjusted limits of agreement between ±31.3% and ±32.7% (mean ±32.0%, SD 0.40). Formulae derived from Japanese, Chinese, Korean, Middle Eastern and Western cohorts, using body surface area, body weight or allometric functions of weight and height, and developed over three decades, therefore differ only in calibration. Rescaling by a single multiplicative constant confirmed this directly, converging all ten to between ±32.4% and ±33.1%, and refitting both slope and intercept gave no further improvement.

Judged on mean bias and the proportion of patients within ±10%, the Lin model performed best among the published formulae, with a mean bias of +5.6%, a mean absolute percentage bias of 12.2%, limits of agreement of ±33.1%, and 56% and 85% of cases within ±10% and ±20% error. The Vauthey-BSA model (+6.9%, 12.9%, ±34.2%, 51% and 84%) and the Poovathumkadavil model (+1.5%, 12.5%, ±33.3%, 50% and 85%) performed comparably. This ranking, however, reflects only which calibration constant happens to suit a white North American cohort, and would not be expected to transfer to a population of different body habitus; the three formulae are equivalent in precision once calibration is accounted for.

The in-house multivariable regression model was developed to test whether additional demographic predictors could break the precision floor observed for the published formulae. In this cohort, it reached limits of agreement of ±29.4% against a floor of approximately ±32%, a relative improvement obtained with five predictors fitted within these data. That improvement is modest and does not alter the clinical conclusion above; we do not propose the model for clinical use, and report it as evidence about the limits of demographic prediction rather than as a tool. A further constraint is that demographic-based models are applicable only within the range of data on which they were developed; extrapolation beyond this range is unreliable. In the training cohort, weight ranged from 42.8 to 137.8 kg, height from 144 to 191 cm, body surface area from 1.40 to 2.63 m^2^, and age from 33 to 96 years, and the in-house model should be regarded as applicable only within these intervals. Because these are the model’s inputs rather than its output, applicability can be assessed for an individual patient before a prediction is made.

Several limitations should be acknowledged. The training cohort consisted of 100 patients of white ancestry treated at a single institution, which limits generalizability and means that seven of the ten formulae evaluated were derived in Asian or Middle Eastern populations and are therefore being applied outside their intended population; part of the calibration offset observed for those formulae may reflect population differences rather than formula quality. Patients had a median age of 70 years with an interquartile range of a single decade, so the estimated age coefficient should not be extrapolated to the younger donors typical of living donor liver transplantation. All patients had stage 2 lung cancer, and a radiologically normal liver is not necessarily equivalent to a healthy liver in a transplant candidate. CT volumetry was treated as the reference standard but is not an error-free measurement of liver volume. Liver contours included the major intrahepatic vessels, a segmentation convention that differs from several of the source studies, and in particular from the autopsy-derived Heinemann formula; some of the observed calibration differences may reflect this rather than true population differences. Error bands, and the ±5% band in particular, should therefore be read as exploratory rather than as evidence that any formula achieves 5% accuracy in an absolute sense. The multivariable regression model was evaluated in a held-out test cohort from the same institution and an earlier time period rather than in an external population; performance in other institutions, imaging protocols, or ancestry groups therefore remains unknown, and external validation would be required before any clinical application could be contemplated.

In summary, within the limits of this exploratory dataset, this work suggests that neither the published demographic SLV formulae nor the in-house model achieve individual-patient agreement adequate for remnant decisions, and generates the hypothesis that residual imprecision, rather than calibration, is the limiting factor in demographic liver volumetry. Clinically, the width of the limits of agreement has direct consequences for patient safety, because the future liver remnant is expressed relative to an estimated total liver volume (FLR/TLV), with the SLV estimate forming the denominator. With limits of agreement between ±25% and ±36% for every model evaluated, a patient whose true FLR/TLV ratio is 25% could be calculated at 19% if SLV were overestimated by 30%, or at 36% if underestimated by the same margin. The first would exclude the patient from a potentially curative resection on the basis of an apparently insufficient remnant; the second would provide false reassurance, permitting resection with an inadequate remnant and an increased risk of post-hepatectomy liver failure. Errors of this magnitude are sufficient to move an individual patient across the 20–25%, 30% and 40% FLR/TLV thresholds in current guidelines. Advances in artificial intelligence for the speed and accuracy of liver segmentation are unlikely to improve SLV modeling directly, since the limitation lies in the demographic predictors rather than in the reference measurement. Because adding predictors, altering the functional form of the size term, and introducing a weight-by-sex interaction all left the residual scatter close to the same floor, future work should focus on including predictors that capture information not contained in body size alone, such as direct measures of body composition and/or liver function.

## Figures and Tables

**Table 2:** Patient demographic data.

Demographic	Training Cohort (*n* = 100)	Test Cohort (*n* = 35)
Age (y): median (Q1–Q3)	70 (65–75)	70 (64–74)
Weight (kg): median (Q1–Q3)	75 (64–84)	74 (66–89)
Height (cm): median (Q1–Q3)	167 (162–173)	165 (159–173)
Sex (Male:Female)	43:57	14:21
BSA, Du Bois (m^2^): median (Q1–Q3)	1.84 (1.70–1.97)	1.83 (1.69–1.99)
BMI (kg/m^2^): median (Q1–Q3)	27 (24–30)	28 (24–30)
CT Liver Volume (mL): median (Q1–Q3)	1466 (1271–1661)	1375 (1148–1770)

**Table 3:** Univariable association with CT-measured liver volume.

Variable	Pearson r	*p*
Weight	+0.811	<0.001
BSA (Du Bois)	+0.813	<0.001
Height	+0.613	<0.001
Sex (male = 1)	+0.452	<0.001
Age	−0.299	0.003

**Table 4:** Cross-validated performance of selected candidate models.

Tier	Model	k	Adj R^2^	CV RMSE (mL)
IV	Weight + height + sex + age + weight × sex (final)	5	0.730	207.9
IV	Quadratic weight + height + sex + age + weight × sex	6	0.733	209.9
III	Quadratic weight + height + sex + age	5	0.725	211.5
IV	Weight + height + sex + weight × sex	4	0.711	214.1
IV	Weight + height + sex + age + weight × height	5	0.714	215.1
II	Weight + height + sex + age	4	0.681	225.3
II	Weight + height	2	0.675	225.9
III	Allometric log–log weight+ height + sex + age	4	0.674	227.4
II	Weight + height + sex	3	0.671	227.8
I	BSA alone	1	0.658	230.5
I	Weight alone	1	0.655	231.4
III	Square-root weight + height + sex + age	4	0.655	234.6

Models are grouped into four prespecified tiers: I, body size alone; II, additive anthropometric terms; III, alternative functional forms of the size term with the Tier II covariates retained; IV, models containing interaction terms. k denotes the number of predictors. Adjusted R^2^ is an in-sample value on the milliliter scale. Cross-validated RMSE (Root Mean Squared Error) were obtained from 200 repetitions of five-fold cross-validation using identical resampling splits for every model, so that values are directly comparable. Rows are ordered by cross-validated RMSE; the final model is shown on top.

**Table 5:** Coefficients of the final model (n = 100, R^2^ = 0.744).

Term	Estimate	SE	t	*p*	95% CI	VIF
Intercept	+197.4	627.32	0.31	0.754	−1048.2 to +1443.0	—
Weight (kg)	+8.68	2.10	4.13	<0.001	+4.51 to +12.84	3.2
Height (cm)	+6.10	3.65	1.67	0.098	−1.15 to +13.35	2.5
Sex (male = 1)	+50.96	58.67	0.87	0.387	−65.5 to +167.5	2.1
Age (years)	−6.14	2.21	−2.77	0.007	−10.54 to −1.74	1.2
(Weight − 77.0) × sex	+11.30	2.65	4.26	<0.001	+6.03 to +16.56	2.5

**Table 6:** Calibration and precision of the eleven SLV models in the training cohort of 100 patients with normal livers. Rows are ordered by mean percentage bias. MAPB, mean absolute percentage bias; LOA, half-width of the 95% limits of agreement. The calibration-adjusted LOA (Equation (4)) is the half-width divided by one plus the mean fractional bias, and removes the dependence of percentage-error spread on calibration; it is reported for all models, since it rescales the observed limits rather than refitting the model. The constant c (Equation (5)) is the recalibration factor, applied identically to every patient; the four columns to its right describe the rescaled prediction c × SLV. Recalibration was not applied to the in-house model, which was fitted to this cohort and is therefore already calibrated to it. The ten published formulae were applied using their published coefficients; metrics for the in-house model were derived from out-of-fold predictions of 200 repetitions of five-fold cross-validation.

Model	Mean Bias (%)	MAPB (%)	95-LOA Half-Width (%)	Within ±5 (%)	Within ±10% (%)	Within ±20% (%)	Calibration Adjusted LOA (%)	c	Bias After Rescaling (%)	LOA After Rescaling (%)	Within ±10% After Rescaling (%)
Chan	−20.3	22.0	±25.3	4	13	46	31.8	1.282	+2.2	±32.4	54
Fu-Gui	−17.7	19.9	±26.5	8	15	51	32.2	1.242	+2.2	±32.9	52
Yuan	−6.9	13.2	±29.5	24	40	78	31.7	1.113	+3.6	±32.8	54
Hashimoto	−6.8	13.2	±29.5	26	42	80	31.7	1.106	+3.1	±32.7	55
Poovathumkadavil	+1.5	12.5	±33.3	24	50	85	32.7	0.997	+1.2	±33.1	51
Lin	+5.6	12.2	±33.1	29	56	85	31.3	0.980	+3.5	±32.4	55
Vauthey-BSA	+6.9	12.9	±34.2	30	51	84	32.0	0.966	+3.2	±33.0	52
Vauthey-weight	+8.4	13.9	±35.1	27	50	79	32.3	0.941	+2.1	±33.0	54
Yu	+10.2	14.6	±35.3	27	46	80	32.0	0.928	+2.3	±32.7	53
Heinemann	+11.3	15.2	±35.5	26	46	74	31.9	0.924	+2.9	±32.8	57
In-house regression model	+1.8	11.1	±29.9	32	58	88	29.4	Not applicable—model fitted to this cohort.			

## Data Availability

The data presented in this study are available on request from the corresponding author.

## Abbreviations

BSA	Body Surface Area
BMI	Body Mass Index
FLR	Future Liver Remnant
FOV	Field of View
GRWR	Graft-to-Recipient Weight Ratio
LOA	Limits of Agreement
NL	Normal Liver
SLV	Standard Liver Volume
TLV	Total Liver Volume
